# Atropine for Critical Care Intubation in a Cohort of 264 Children and Reduced Mortality Unrelated to Effects on Bradycardia

**DOI:** 10.1371/journal.pone.0057478

**Published:** 2013-02-28

**Authors:** Peter Jones, Mark J. Peters, Nathalia Pinto da Costa, Tobias Kurth, Corinne Alberti, Katia Kessous, Noella Lode, Stephane Dauger

**Affiliations:** 1 Critical Care Group - Portex Unit, Institute of Child Health, University College London, London, Great Britain; 2 Réanimation Pédiatrique, Assistance Publique-Hôpitaux de Paris, Hôpital Robert Debré, Paris, France; 3 Institut National de la Sante et de la Recherche Medical, Bordeaux, France; 4 Neuroepidemiology, University of Bordeaux, Bordeaux, France; 5 Division of Preventive Medicine, Brigham and Women’s Hospital, Harvard Medical School, Boston, Massachusetts, United States of America; 6 Unité d’Epidémiologie Clinique, Assistance Publique-Hôpitaux de Paris, Hôpital Robert Debré, Paris, France; 7 Institut National de la Sante et de la Recherche Medical CIE 5, Hôpital Robert Debré, Paris, France; 8 Université Paris Diderot, Paris, France; Nottingham University, United Kingdom

## Abstract

**Background:**

Atropine has is currently recommended to facilitate haemodynamic stability during critical care intubation. Our objective was to determine whether atropine use at induction influences ICU mortality.

**Methodology/Principal Findings:**

A 2-year prospective, observational study of all first non-planned intubations, September 2007–9 in PICU and Intensive Care Transport team of Hôpital Robert Debré, Paris, 4 other PICUs and 5 NICUs in the Paris Region, France. Follow-up was from intubation to ICU discharge. A propensity score was used to adjust for patient specific characteristics influencing atropine prescription. 264/333 (79%) intubations were included. The unadjusted ICU mortality was 7.2% (9/124) for those who received atropine compared to 15.7% (22/140) for those who did not (OR 0.42, 95%CI 0.19–0.95, p = 0.04). One child died during intubation (1/264, 0.4%). Two age sub-groups of neonates (≤28 days) and older children (>28 days, <8 years) were examined. This difference in mortality arose from the higher mortality in children aged over one month when atropine was not used (propensity score adjusted OR 0.22, 95%CI 0.06–0.85, p = 0.028). No effect was seen in neonates (propensity score adjusted OR 1.3, 95%CI 0.31–5.1 p = 0.74). Using the propensity score, atropine maintained the mean heart rate 45.9 bpm above that observed when no atropine was used in neonates (95%CI 34.3–57.5, p<0.001) and 43.5 bpm for older children (95%CI 25.5–61.5 bpm, p<0.001).

**Conclusions/Significance:**

Atropine use during induction was associated with a reduction in ICU mortality in children over one month. This effect is independent of atropine’s capacity to attenuate bradycardia during intubation which occurred similarly in neonates and older children. This result needs to be confirmed in a study using randomised methodology.

## Introduction

Critical care intubation (CCI) of young children in critical care situations represents a crucial moment in an episode of critical illness and may be associated with cardiorespiratory decompensation. In critically-ill adults the risk of death during CCI has been estimated at 1–3% with pre-existing hypotension greatly increasing this risk. [1], [2] In children intubation mortality is lower. No deaths were observed in either of two retrospective studies of 137 intubations in 103 children outside the Operating Room [3] and or 143 children in the Emergency Department [4].

Paediatric anaesthetists and intensivists have used slowing of the heart rate in children as a measure of haemodynamic stability during CCI for more than 50 years. Two distinct mechanisms contribute to falls in heart rate during intubation; the direct effects of some induction drugs, and vagally-mediated parasympathetic cholinergic inhibition of sino-atrial node activity as a reflex response to hypoxaemia and/or laryngeal stimulation [5].

Atropine was proposed in the 1950s to overcome the anti-cholinergic effects of suxamethonium during induction and intubation. [6] Its efficacy in attenuating falls in heart rate during anaesthetic intubation in children is proven. [7], [8] Following the introduction of non-depolarising muscle relaxants and less cardio-depressant anaesthetics, such as sevoflurane, there has been a decline in atropine use [9].

Atropine is currently recommended to optimise haemodynamic stability during CCI. [10] The 2007 American College of Critical Care Medicine clinical practice parameters for hemodynamic support in paediatric septic shock provide a level III (expert opinion) recommendation for “*Ketamine and atropine pretreatment …[to] be used as an …induction regimen of choice to promote cardiovascular integrity”.* [11] The lack of prospective data on the impact of atropine on heart rate and outcome in critically ill children prompted this study.

Our objective was to measure the effect of atropine use on ICU mortality in a prospectively-recruited population of children undergoing their first CCI. We assumed the null hypothesis that atropine had no effect on either outcome. A propensity score was used to adjust for patient specific prognostic characteristics that might have influenced atropine prescription.

## Methods

### Study Design and Population

The study was multi-centre, prospective, observational and used a propensity score to adjust for atropine use. An information letter was provided for parents describing the objective of the study and giving them the possibility of removing their child from the study. The process was not documented. Written consent was not obtained as the study did not involve intervention to the process of intubation. This procedure is in accordance with French law and was approved by the Comité d’Ethique et d’Evaluation en Recherche Biomédicale of the Groupe Hospitalo-Universitaire-Paris Nord.

The ECG recordings were made in the Paediatric Intensive Care Unit (PICU) and by the Paediatric/Neonatal Intensive Care Transport Service (ICT) of the l’Hôpital Robert Debré, Paris, France, between September 2007 and September 2009. Children were intubated by the ICT Team and transported to four participating PICUs (Robert Debré, Kremlin-Bicêtre, Necker and Trousseau Hospitals) and five Neonatal ICUs (Robert Debré, Creteil, Institut de Puériculture, Montreuil and Port Royal Hospitals) in the Paris Region. Intubation ‘for transport’ is not practised by the ICT Team.

All children who were undergoing their first non-elective intubation and were not asystolic were eligible for inclusion. The hospital protocol was not to give atropine routinely to children aged over eight years, hence such cases were excluded. The following data were prospectively recorded: age, sex, gestational age (for births), pathology (neonatal respiratory distress [NRD], non-neonatal respiratory distress [non-NRD], cardiac, neurological, ear nose and throat [ENT], sepsis and ‘other’), principal induction drug and atropine administration. The hospital protocols for intra-venous intubation drugs were; atropine 20 mcg/kg, etomidate 0.4 mg/kg, propofol 2–4 mg/kg, ketamine 1–2 mg/kg, thiopental 5 mg/kg, morphine 0.1 mg/kg, midazolam 0.1 mg/kg, sufentanyl 0.2 mcg/kg, suxamethonium 2 mg/kg less than 18 months and 1 mg/kg more than 18 months, vercuronium 0.1 mg/kg. The PRISM III-24 (Paediatric RISk of Mortality) score was recorded in the children >28 days [12].

Prescription of atropine was at the discretion of individual intensivists. Those who received atropine were designated the atropine group and comparisons were made to the no-atropine group. Children who received atropine *after* the start of the intubation as treatment of bradycardia were included in the no-atropine group on the basis of the intention-*not*-to-treat.

The study population was divided *a priori* into two age sub-groups, neonatal (≤28 days post-natal age) and older children (>28 days post-natal age and <8 years), for the purposes of analysis. The rationale to divide the group at this age was due to the physiological transition in autonomic innervation that occurs during early extra-uterine life [13] and due to the different distribution of pathologies in the two groups.

### Study Procedures

A one-minute ECG control strip (25 mm/s) was recorded prior to the start of intubation, and used to calculate the mean pre-intubation heart rate by averaging the lowest and highest heart rate.

Continuous ECG/SpO_2_ recording started from the moment of insertion of the laryngoscope until a SpO_2_ of ≥95% had been obtained or the child connected to a ventilator. Two Paediatric Intensivists reviewed the intubation ECGs independently and an average was made of the two lowest, consecutive R-R intervals. The number of attempts at intubation was recorded on the ECG by the intensivist.

### Propensity Score Construction

The propensity score (PS) is a one-dimensional summary of multidimensional covariates that is used to make causal inferences in observational studies by dealing with confounding patient-specific prognostic characteristics that could have an effect on both treatment assignment and outcome. The PS for a group of patients is the sum of the probabilities for having received a particular treatment according to a specified set of pre-treatment covariates that might plausibly have affected treatment assignment [14], [15].

We constructed a PS for atropine prescription using the pre-prescription variables (Table 1, with the exception of the PRISM score) which included 10 or more children that had been identified by a group of 61 Paediatric Intensivists in eight countries on three continents as influencing their decision to prescribe atropine. [16] Linearity in the log odds of continuous variables was assessed visually and by using likelihood ratio testing.

**Table 1:** Population characteristics for all patients and the two age sub-groups of neonates and older children.

	All Patients (n = 264)		Neonates (n = 153)		Older children (n = 111)	
	No-atropine n = 140(%)	Atropine n = 124(%)	No-atropine n = 74(%)	Atropine n = 79(%)	No-atropine n = 66(%)	Atropine n = 45(%)
Median age days [IQR]	21* [0–220]	1* [0–71]	0 [0–2]	0 [0–1]	225 [78–861]	134 [67–325]
Boys	94 (67)	85 (69)	56 (76)	60 (76)	38 (58)	25 (56)
Mean baseline heart rate, min-1 (SD)	157* (133–181)	150* (125–175)	154 (132–176)	148 (126–170)	161 (135–187)	154 (124–184)
NRD	56 (40)	61 (49)	56 (76)	61 (77)	–	–
NonNRD	40 (29)	18 (15)	8 (11)	4 (5)	32 (48)	14 (31)
Cardiac	9 (6)	4 (3)	4 (5)	3 (4)	5 (8)	1 (2)
ENT	9 (6)	11 (9)	1 (1)	1 (1)	8 (12)	10 (22)
Neurologic	13 (9)	20 (16)	1 (1)	5 (6)	12 (18)	15 (33)
Sepsis	10 (7)	7 (6)	1 (1)	3 (4)	9 (14)	4 (9)
Other	3 (2)	3 (2)	3 (4)	2 (3)	0 (0)	1 (2)
No sedation drugs	35 (25)	37 (30)	30 (41)	35 (44)	5 (8)	2 (4)
Ethomidate	5 (4)	5 (4)	0 (0)	1 (1)	5 (8)	4 (9)
Propofol	44 (31)	23 (19)	10 (14)	2 (3)	34 (52)	21 (47)
Ketamine	12 (9)	3 (2)	0 (0)	0 (0)	12 (18)	3 (7)
Morphine	17 (12)	12 (10)	15 (20)	11 (14)	2 (3)	1 (2)
Midazolam	12 (9)	22 (18)	6 (8)	15 (19)	6 (9)	7 (16)
Sufentanyl	14 (10)	19 (15)	13 (18)	15 (19)	1 (2)	4 (9)
Other	1 (1)	3 (2)	0 (0)	0 (0)	1 (2)	3 (7)
Suxamethonium	8 (6)	3 (2)	0 (0)	0 (0)	8 (12)	3 (7)
Vecuronium	1 (1)	3 (2)	1 (1)	0 (0)	3 (5)	0 (0)
					No-atropine (n = 57/66)	Atropine (n = 37/45)
PRISMn = 93	–	–	–	–	10.5 [7]–[19]	9 [5–13.5]

All of the variables from the columns ‘All Patients’ were entered into the propensity score. the PRISM score was not used for the propensity score. Neonates are ≤28 days and older children >28 days and less than 8 years.

* p value <0.05 for the difference between the atropine and non-atropine groups.

NRD - neonatal respiratory distress, Non-NRD - non-neonatal respiratory distress, ENT - ear nose and throat, PRISM - Paediatric RISk of Mortality score.

### End Points

Our primary end point was ICU mortality. The secondary end points were the difference in heart rate from pre-intubation baseline to the lowest two R-R complexes during intubation and the number of days from intubation to death.

### Statistical Analysis

Qualitative variables are described as numbers and percentages and quantitative variables as median [quartiles] or mean (standard deviation) according to their Gaussian distribution. Independent t-tests or a Mann-Whitney test were used for continuous data according to their distribution and Fisher’s exact test was used for categorical data. All statistical tests were 2-sided and the probability of a type 1 error (α) was determined at <0.05. Univariate and/or multivariable linear or logistic regression models were constructed to test the effect of atropine using the PS. Effect estimates were calculated for numerical variables and odds ratios (OR) for categorical variables with their corresponding 95% confidence intervals (95%CI). Pearson correlation was used to compare quantitative variables. Kaplan-Meier plots and Log-rank tests were used for survival analysis. Missing data were excluded list-wise. Statistical tests were carried out using SPSS (version 19) with the exception of the PS which was performed using SAS V.9.2 software (SAS, Cary, NC).

## Results

### Study Patients and Atropine Use

**Figure 1:**
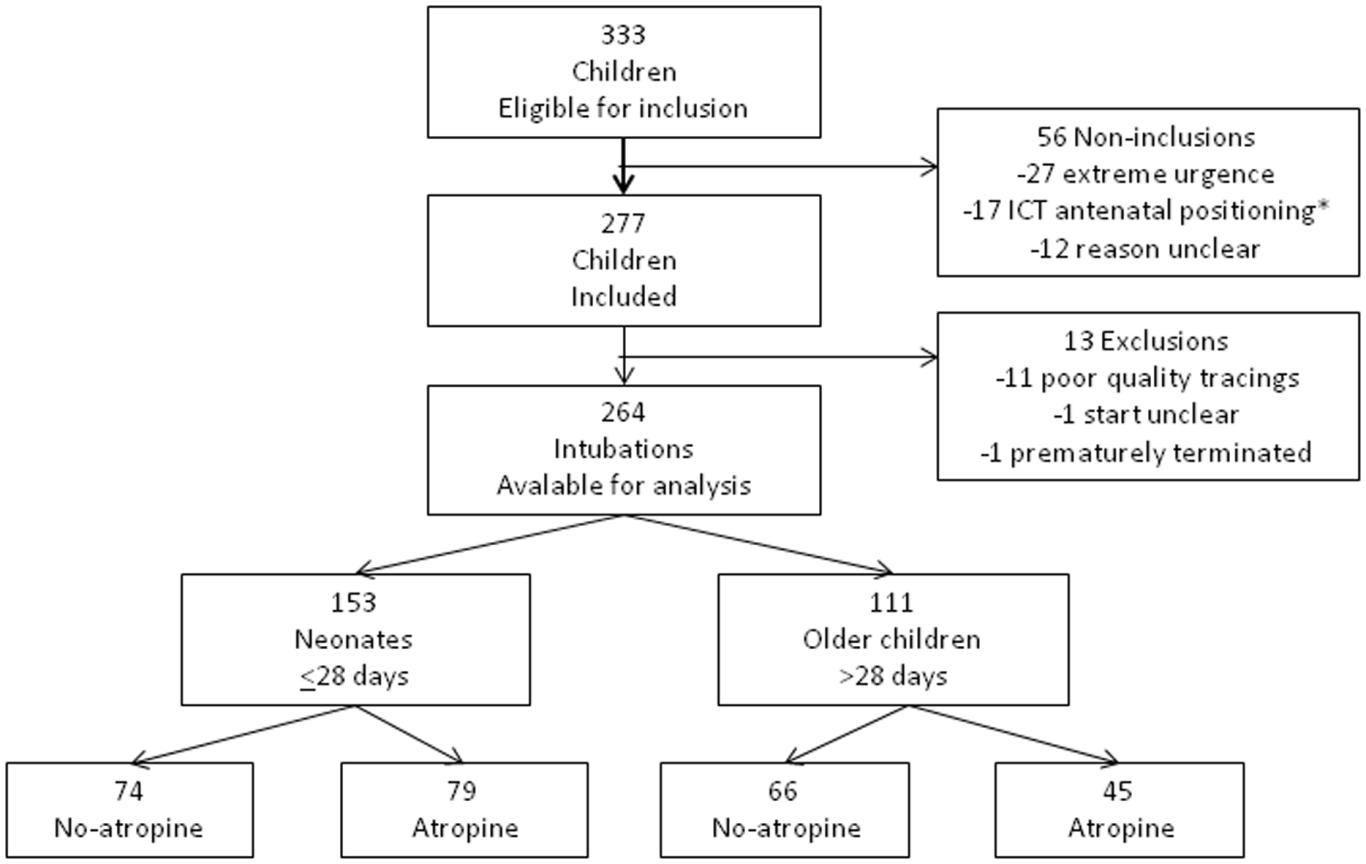
Flow-chart of inclusions, non-inclusions and exclusions. Neonates are ≤28 days and older children >28 days and less than 8 years. *ICT - Intensive Care Transport team positioned antenatally for premature births.

A total of 333 children were eligible for inclusion of which 277 were included. Fifty-six children were not included and 13 children were excluded (Figure 1). A total of 264 study patients were available for analysis (264/333, 79% [95%CI 75–83]) of which 114 from PICU and 150 from ICT. Twenty-three intensivists included a median of 5 [1]–[16] children each. Atropine was prescribed in 124 of the 264 (47% [95%CI 41–53]) intubations. It was more frequently prescribed, although not significantly (p = 0.08), during the intubations of neonates (79/153, 52% [95%CI 44–59]) than older children (45/111, 41% [95%CI 32–50]) (Table 1 and Figure 1). The median frequency of atropine prescription per intensivist was 2 [1]–[8]. The PRISM score was retrospectively available for 94 of 111 older children and was not significantly (p = 0.09) different between the atropine and non-atropine groups (Table 1).

### Crude ICU Mortality

Thirty-one children died during ICT/ICU care (31/264, 12% [95%CI 8–16]). There was significantly lower mortality (p = 0.003) in the neonatal group 7% (10/153 [95%CI 4–12]) than for the older children 19% (21/111 [95%CI 13–27]). No children were lost to follow up. Only one child died during intubation (aged 16 months with a lethal chromosomal disorder, pulmonary hypertension and septic shock). This represents an overall 0.4% [95%CI 0.07–2.1] mortality for the immediate intubation period.

**Figure 2:**
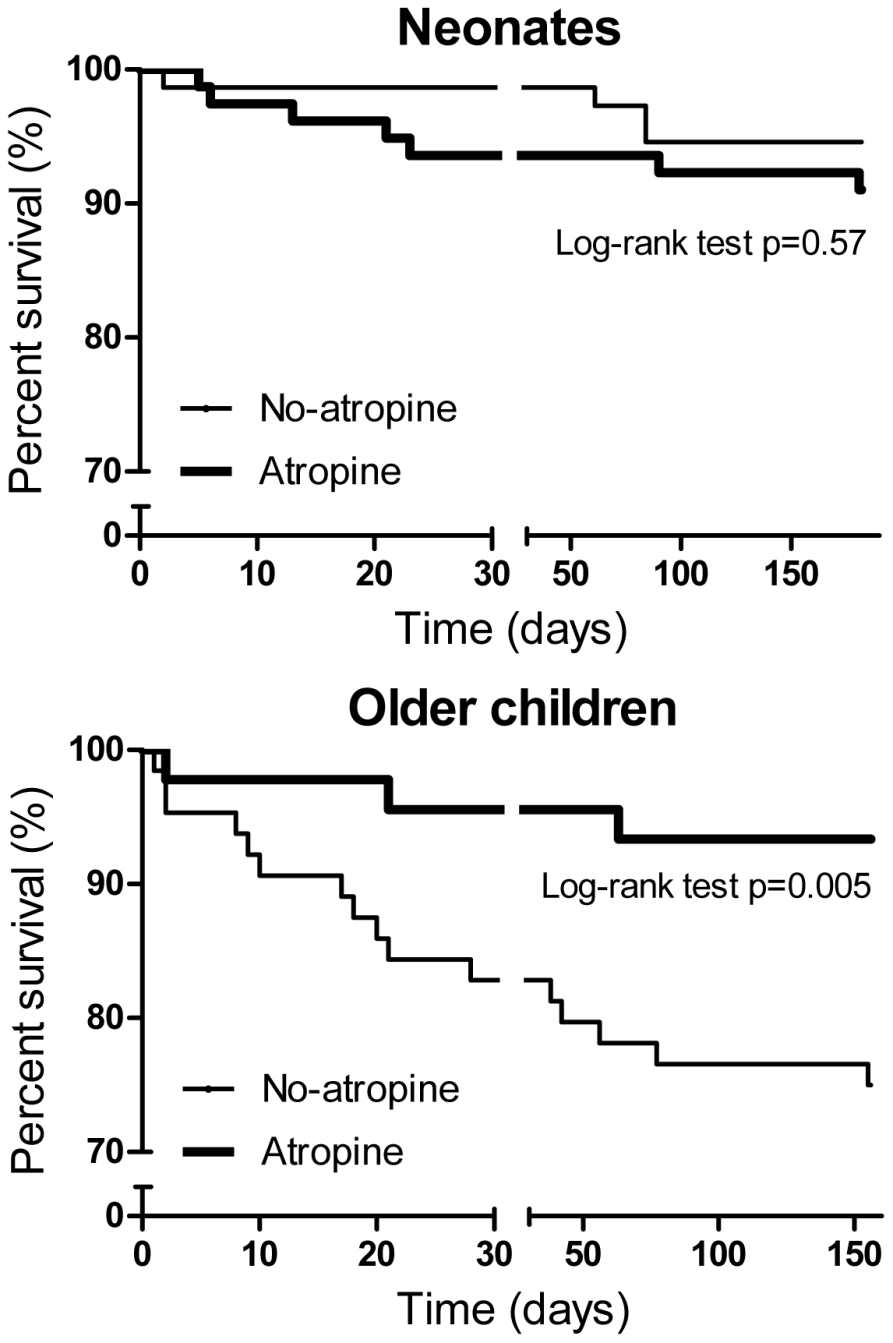
Kaplan-Meier plots for mortality in the two age sub-groups of neonates and older children.

The unadjusted mortality prior to PICU discharge was 7% (9/124 [95%CI 4–13]) for those who received atropine compared to 16% (22/140 [95%CI 11–23]) for those who did not (OR 0.42, 95%CI 0.19–0.95 p = 0.04). The median mortality estimated by using the PRISM score was 8% [3]–[30] without atropine and 5% [2]–[12] with atropine. The standardised mortality ratio (SMR) between using the PRISM score as predictor of mortality (for the 94/111children for whom the score was available) was 1.05 for the whole group and 1.16 for the no-atropine *versus* 0.74 for the atropine groups. Ethomidate was used for 10 intubations, five with and five without atropine. All the children who received atropine survived with three deaths amongst those who did not receive atropine. Atropine use was associated with a significant difference in the survival of the older children (p = 0.005) but not neonates (p = 0.57) (Figure 2). The excess mortality present in older children with non-neonatal respiratory, cardiac and septic shock categories (Table 2).

**Table 2:** Breakdown of the causes of death by pathology. Neonates are ≤28 days and older children >28 days and less than 8 years.

	Neonates (n = 153, deaths/cases)		Older children (n = 111, deaths/cases)	
	Non-atropine n = 74(n%)	Atropine n = 79(n%)	Non-atropine n = 66(n%)	Atropine n = 45(n%)
Neonatal respiratory distress	1/56(2)	2/61 (3)	–	–
Non-neonatal respiratory distress	2/8 (3)	0/4 (0)	9/32 (14)	0/14 (0)
Cardiac	0/4 (0)	0/3 (0)	2/5 (3)	0/1 (0)
Ear, nose and throat	1/1 (1)	0/1 (0)	1/8 (2)	0/10 (0)
Neurologic	0/1 (0)	3/5 (4)	3/12 (5)	3/15 (7)
Sepsis	0/1 (0)	1/3 (1)	3/9 (5)	0/4 (0)
Other	0/3 (0)	0/2 (0)	0/0 (0)	0/1 (0)
**Total**	**4** (5)	**6** (8)	**18** (27)	**3** (7)

### Construction of the Propensity Score and Adjusted ICU Mortality

Only two of the variables of baseline pre-atropine prescription characteristics were significantly different between the atropine and no-atropine groups, these were the median age of patients (p = 0.02) and the mean baseline heart rate (p = 0.02). When the two age sub-groups were further examined, there was no significant difference between any of the variables. Three variables were not included in the construction of the PS because they featured in less than ten cases: fentanyl (n = 1), thiopental (n = 4) and vecuronium (n = 4). The c-statistic of the PS was 0.72.

When the effect of atropine on mortality was examined in the age subgroups using the PS there was a significant reduction of mortality in the older children, but not in the neonates (Table 3). We conducted a *post hoc* analysis to consider the apparent association of atropine treatment with death by using two PS constructed from the variables of each age group. This was done to test the possibility that the construction of the PS from data from all children would be different if the PS was constructed using data from children of each age group. The association of atropine use with death remained relatively unchanged: *post hoc* PS adjusted OR in neonates of 2.4, (95%CI 0.56–10.4, p = 0.23) and *post hoc* PS adjusted OR 0.24 (95%CI 0.06–0.96, p = 0.043) for the older children (see Table 3).

**Table 3:** ICU mortality related to atropine use corrected by the propensity score (PS) in the two age subgroups. Neonates are ≤28 days of age and older children >28 days and less than 8 years.

	Neonates (n = 153)			Older children (n = 111)		
	Odds ratio	P	CI 95%	Odds ratio	p	CI 95%
Atropine crude mortality	1.4	0.59	0.39, 5.3	0.21	0.017	0.06, 0.75
Atropine PS corrected	1.3	0.74	0.31, 5.1	0.22	0.028	0.06, 0.85

### Details of the Intubations

**Figure 3:**
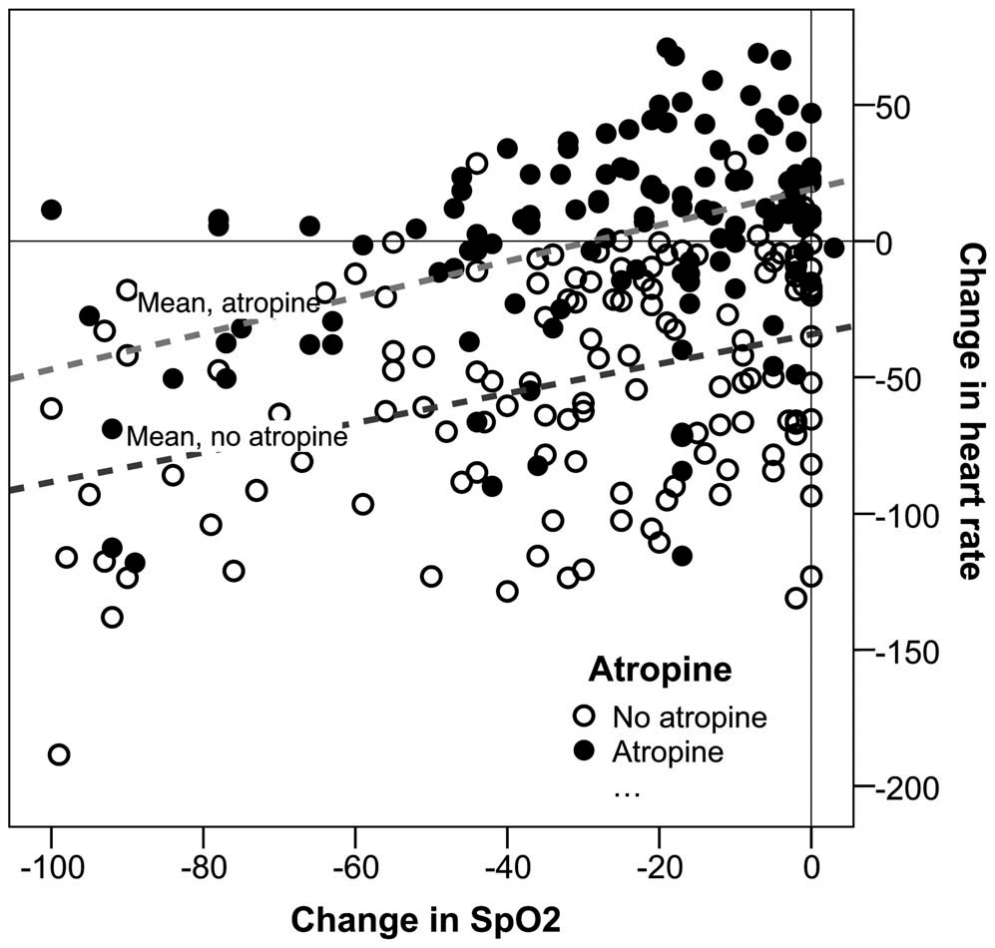
The change in heart rate related to a change in peripheral oxygen saturation (SpO_2_) during intubation.

Measured oxygen saturations were similar between atropine and non-atropine groups. Median fall in SpO_2_ was 25% [8–44] in the non-atropine group and 19% [6–44] in the atropine group (p = 0.31). There was a significant correlation between a fall in SpO_2_ and a fall in heart rate (p<0.0001) (Figure 3 and Table 4). The median number of attempts at intubation was also comparable between non-atropine (1 [1]–[2]) and atropine (1 [1]–[2]) (p = 0.20) cases. One attempt at intubation significantly reduced mean heart rate in the neonates by 9.6 beats-per-minute (bpm) (95%CI 2.3–16.9, p = 0.01) but not in the older children (6.1 bpm per intubation attempt, 95%CI 1.5–13.8, p = 0.12). Eleven children were excluded from the saturation analysis and 16 from the number of attempts at intubation because of missing data.

**Table 4:** Multivariable analysis of the change in heart rate during intubation following fall in peripheral oxygen saturation (SpO_2_) and after the use of atropine (corrected by the propensity score [PS]). Neonates are ≤28 days and older children >28 days and less than 8 years.

	Neonates (n = 153)			Older children (n = 111)		
	Heart rate change (bpm)	P	CI 95%	Heart rate change (bpm)	p	CI 95%
Fall in SpO_2_ by 10% points	−4.1	0.005	−1.3, −7.0	−5.4	<0.001	−2.6, −8.4
Difference with and without atropine (PS corrected)	−45.9	<0.001	−34.3, −57.5	−43.5	<0.001	−25.5, −61.5

### Adjusted Change in Heart Rate

**Figure 4:**
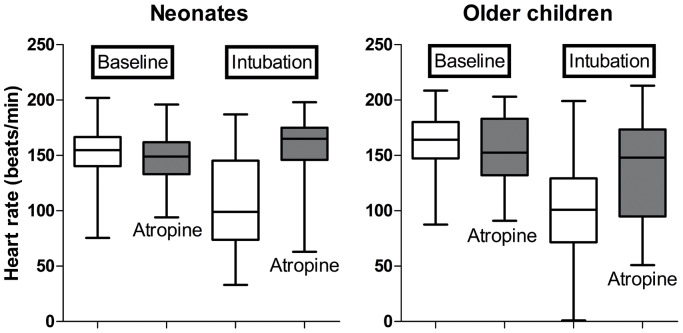
The influence of atropine of the change in heart rate during intubation of the two age sub-groups.

The use of the PS had little effect on the crude estimates of the mean change in heart rate (Figure 4) which was 58.7 bpm without the PS *versus* 53.4 bpm with the PS for the neonates and 41.5 bpm without the PS *versus* 40.8 bpm with the PS for the older children. The PS adjusted changes in heart rate due to changes in SpO2 and attempts at intubation are shown in Table 4.

## Discussion

We disproved our hypothesis and showed that the use of atropine was associated with a lower overall ICU mortality. This result is unrelated to any effect on intubation bradycardia which occurred in a similar measure in both age groups when atropine was not used.

Our population was diverse with 57% of children being intubated and hospitalised outside the PICU of Robert Debré. Atropine was used in 47% of our cases. This is similar to a 2004 survey in 204 French PICUs [17] and a 2010 report from UK neonatal units. [18] A recent survey of influences on atropine use and atropine prescribing habits among 61 paediatric intensivists in 8 countries noted that 49% prescribed atropine for half of their intubations. [16] One area where our practice differs from others is the relatively infrequent use of muscle relaxants including suxamethonium. This is due to several reasons; firstly the large proportion of neonates for whom our hospital protocol does not recommend muscle paralysis, secondly, a relatively large proportion of ENT intubations and finally the high proportion of propofol use which is associated with good intubation conditions without relaxants [19].

When we compare the intubation conditions in our study population with published series, the number of intubation attempts were equivalent to existing neonatal [20], [21] and paediatric studies. [3], [4] Similarly, median trough SpO_2_ values for the neonates (76%) were consistent with previously published values (58–80%). [18], [20], [21] In PICU Carroll *et al.* [3] noted that peripheral desaturation below 90% occurred in 29% of intubations (40/137) and in the Emergency Department, Fastle and Roback recorded an incidence of 22% (31/143). [4] If we use an identical definition for our study population, as many as 75% of the children were desaturated. Nevertheless, neither of these studies recorded median fall in saturation and both reflected on the bias inherent in their retrospective, self-reporting methodologies.

The mortality in our intubated study population of older children was 19% (SMR 1.05 using the PRISM score as a mortality predictor) which compares to 8.3% mortality for emergency admissions and 3.7% overall mortality in PICU for all ages during the study period. This contrasts to 4.8% PICU mortality for children under 10 years in Great Britain and Ireland for the years 2007–9 [22].

The propensity score is a valuable tool for analysing differences in drug effect in non-randomised studies where selection bias may be important. [23] We chose a multivariable regression based model which provides more statistical power. [24] Our study design produced treatment groups of neonates and older children where there were no significant differences between pre-treatment variables (Table 1). This validates the use of the PS for which a considerable overlap in the distribution of variables is an *a priori* condition. In addition, our result for the crude OR for the ICU mortality in the older children (0.21) remained stable when adjusted using an all-patients PS (0.22) and when using a *post hoc* older children PS (0.24). Despite our result remaining significant when using a PS for all patients and divided for the two age groups, our findings need to be confirmed in a study using randomised methodology.

There are three possible explanations for the unexpected significant difference in ICU mortality with and without atropine. Firstly, the PS has not dealt with atropine prescription bias because not all prescription-influencing co-variates were included in its construction, which is a condition of its use. [14] This means that residual confounders remained after using the PS which linked atropine prescription to death. A possible indication of this could be the non-significant difference in the PRISM scores and SMR of the atropine and non-atropine groups, which showed a trend towards an increased risk of death for the non-atropine group. The significant difference in the baseline heart rate between the two groups is integrated in the PRISM score but was also included in the PS before adjusting the final result. Secondly, that chance has produced a statistically significant result and finally that the effect of atropine in reducing mortality is real.

The excess mortality we observed was not related to mortality during intubation because this was rare with only a single death observed. Neither can the excess post-intubation mortality be ascribed to a late effect of bradycardia because this occurred with similar frequency in both the neonates and older children (Table 4 and Figure 4) yet the ICU mortality difference was only seen in the older children.

One possible explanation for the observed effect of atropine on mortality may be related the uncoupling of real-time feedback of measurements of blood pressure in the baroreceptors in the carotid bodies, which has the effect of reducing R-R beat-to-beat variability (RRBBV). Vagal impulses are rapid by nature of the rapid hydrolysation of acetylcholine in postganglionic parasympathetic receptors contrary to sympathetic stimulation of the heart and vessels which is of the order of 2–5 seconds. [25] Reductions in RRBBV following atropine administration have been measured in premature neonates awaiting intubation for respiratory distress [26] and normal children undergoing routine anaesthesia. [27] In small children, reductions in RRBBV could be achieved by a single dose of atropine due to its long half-life [28] which is a consequence of the extended elimination phase resulting from its increased distribution volume [29].

Pre-terminal haemodynamic decompensation is frequently accompanied by bradycardia in children. [30] It results from a ‘two-hit’ process whereby the first step is often exaggerated RRBBV before the second step of entry into a negative feedback loop of arrhythmia and hypoperfusion, as has been demonstrated experimentally in dogs. [31] The short-lived intubation bradycardia commonly observed in our non-atropinised patients is an example of severe RRBBV, yet intubation mortality was rare because the children were still self-protecting from the second step of bradycardia-hypoperfusion. Atropine is ineffective against hypo-perfusion and non-vagally mediated bradycardia when they are already established, indeed atropine is no longer recommended by the AHA for resuscitation. [32] However, when atropine is prophylactically administered before bradycardia-hypoperfusion, as happened to our atropinised older patients, some of whom later became vulnerable to haemodynamic decompensation, atropine could protect against the first step of spontaneously occurring exaggerated RRBBV.

Despite the prolonged half-life of atropine in the young, as mentioned above, the protection accorded is unlikely to be responsible for the entirety of the reduction observed in mortality in the older children because the many of these deaths occurred more than several days after intubation (Figure 2).

### Conclusions

Atropine was associated with a reduction in ICU mortality outside the immediate period of the intubation only in children aged over one month. This effect is independent of its capacity to reduce heart slowing during intubation. The association between atropine use during CCI and reduced ICU mortality should be investigated in other populations using a randomised methodology.
